# The application effect of mobile internet technology in the postoperative follow-up management of patients with lumbar intervertebral disc protrusion

**DOI:** 10.3389/fsurg.2026.1876447

**Published:** 2026-08-31

**Authors:** Yan Ruan, Danlei Zheng, Weiwei Chu, Jie Yu

**Affiliations:** Department of Nursing, The Fourth Affiliated Hospital of Zhejiang University School of Medicine, Yiwu, China

**Keywords:** aftercare, lumbar disc herniation, mobile applications, patient compliance, self-management, telemedicine

## Abstract

**Objective:**

To explore the application effect of mobile Internet technology in the postoperative follow-up management of patients with lumbar intervertebral disc protrusion.

**Methods:**

100 patients with lumbar disc herniation undergoing percutaneous lumbar discectomy were randomly divided into two groups. The control group (50 cases) received routine follow-up management, and the intervention group (50 cases) received follow-up management based on mobile Internet technology. The score of self-management ability, the score of the Japanese Orthopaedic Association (JOA) Lumbar Function Scale, the score of the functional exercise compliance scale of orthopaedic patients, the rate of good and good rehabilitation and the rate of recurrence were compared between the two groups.

**Results:**

Repeated-measures ANOVA revealed significant time × group interaction effects for self-management ability (*F* interaction = 14.276, *P* < 0.001), JOA score (*F* interaction = 13.543, *P* < 0.001), and rehabilitation exercise adherence (*F* = 10.132, *P* < 0.001), indicating that the intervention group experienced a significantly slower decline in rehabilitation exercise adherence and showed greater improvements in self-management ability and JOA scores over time. At 3 months post-discharge, the intervention group showed a significantly higher excellent rehabilitation rate (92.00% vs. 76.00%, *P* < 0.05) and was associated with a lower short-term recurrence rate (0.00% vs. 10.00%; Fisher's exact test, *P* = 0.056), although this difference did not reach statistical significance. The potential for reducing recurrence risk warrants further investigation in adequately powered studies with extended follow-up periods. Furthermore, logistic regression analysis identified the mobile Internet-based intervention as the strongest independent predictor of excellent recovery (adjusted OR = 8.42, 95% CI: 2.75–25.78, *P* < 0.001). Multiple linear regression models confirmed that the intervention was significantly associated with higher scores in self-management behavior (*β* = 0.712, *P* < 0.001), exercise adherence (*β* = 0.694, *P* < 0.001), and lumbar function (*β* = 0.654, *P* < 0.001) at 3 months.

**Conclusion:**

Follow-up management based on mobile Internet technology significantly enhances postoperative self-management ability, promotes lumbar function recovery, and significantly mitigates the decline in rehabilitation exercise compliance in patients with lumbar disc herniation. At 3 months post-discharge, the intervention was associated with a lower short-term recurrence rate (0.00% vs. 10.00%), but this difference did not reach statistical significance by Fisher's exact test (*P* = 0.056), and the study was not powered to detect recurrence differences. Longer-term follow-up in larger samples is required to determine whether the intervention confers a sustained reduction in recurrence risk. Regression analyses confirm that this intervention is a strong independent factor contributing to these superior outcomes. However, as both participants and providers were not blinded to group assignment, the observed effects may be partially attributable to increased attention and contact time rather than the digital intervention alone.

**Clinical Trial Registration**: ClinicalTrials.gov, identifier NCT07547410.

## 1 Introduction

Lumbar disc herniation (LDH) is a complex condition manifested by irritation or compression of nerve roots and cauda equina nerve due to disc deformation, rupture of the fibrous ring, and herniation of the nucleus pulposus. The most common symptoms include low back and leg pain, tingling, sensory disturbances, and abnormal lower extremity muscle strength, and are one of the leading causes of disability worldwide, with a recurrent course (1). According to statistics from the Ministry of Health, there are currently more than 200 million LDH patients in China, accounting for 15.2% of the country's total population, and the incidence rate in men is higher than that in women (2). At present, about 87% of LDH patients can be relieved by conservative treatment, but surgery is the most effective treatment method for patients who are ineffective to conservative treatment and have persistent low back and leg pain or progressive deterioration of neurological dysfunction, and patients need to undergo rehabilitation training for a long time after surgery to ensure the efficacy of surgery (3).

Long- term clinical studies have found that about 60% of LDH patients have postoperative lumbar spine function decline, residual low back and leg pain, and even 5%–11% of patients need to undergo surgery again (4). The reasons for this are related to the lack of self-management ability of most patients, poor coordination and compliance with rehabilitation exercises, which is also one of the important factors leading to the recurrence of LDH after surgery (5). In addition, the routine out-of-hospital follow-up management measures only stay at the level of telephone follow-up and outpatient review, which cannot achieve long-term effective management of patients. In recent years, mobile Internet technology has gradually emerged in the medical and nursing fields, which can effectively improve the follow-up experience and follow-up efficiency (6).

Mobile health (mHealth), defined by the World Health Organization as medical and public health practice supported by mobile devices, encompasses a broad spectrum of applications including telemonitoring, patient education, decision support, and behavior change interventions (7). In the context of postoperative follow-up, mHealth technologies offer distinct advantages over traditional telephone-based or outpatient-only models: they enable real-time bidirectional communication between patients and providers, support automated behavior monitoring and feedback, deliver multimedia educational content tailored to individual recovery stages, and generate longitudinal data that can inform clinical decision-making. The core mechanisms through which mHealth interventions are theorized to improve self-management outcomes include enhanced self-efficacy via mastery experiences (patients successfully completing prescribed exercises with remote guidance), improved knowledge retention through multimedia repetition, and sustained behavioral reinforcement through automated reminders and social support features (8, 9).

In this context, follow-up management is conceptualized as a structured, proactive, and multidisciplinary clinical service process that extends clinical care beyond hospital discharge, encompassing continuous monitoring, reinforcement of rehabilitation behaviors, early detection of complications, and bidirectional communication. Unlike traditional passive follow-up, this proactive model operates on the principle that recovery requires iterative assessment and personalized adjustment. The mobile Internet technology employed in this study refers to the technical architecture that enables this process, including smartphone-based platforms, cloud computing, and intelligent algorithms (e.g., AI pose estimation). While mHealth constitutes the broader application domain encompassing behavioral, educational, and policy dimensions—mobile Internet technology provides the operational backbone that makes proactive follow-up management scalable and efficient. The specific platform implementation (a WeChat mini-program) serves as the user-facing interface that operationalizes these technical capabilities. It encompasses three core functional domains: (1) real-time bidirectional data exchange between patients and care teams; (2) automated, protocol-driven behavioral monitoring and feedback (e.g., daily check-in, AI-assisted exercise form analysis); and (3) intelligent data processing and risk stratification to enable proactive, personalized care coordination. While mHealth constitutes the broader application domain—including the behavioral, educational, and policy dimensions of mobile-based healthcare—mobile Internet technology provides the technical architecture and operational backbone that enables mHealth interventions to function at scale, with the specific platform implementation (e.g., WeChat mini-program, native app) serving as the user-facing interface. In the present study, the mobile Internet-based follow-up system operationalizes these principles by integrating multimedia education, AI-powered movement monitoring, automated reminders, and risk-stratified supervision into a unified platform, thereby transforming the traditional postoperative follow-up model from a reactive, schedule-driven process into a proactive, data-driven, and patient-centered care continuum.

However, despite these theoretical advantages, the empirical evidence base for mHealth in post-spinal surgery rehabilitation remains nascent, with most existing studies limited by short follow-up periods, small sample sizes, and heterogeneous intervention designs (10).

Although several studies have examined mobile-based rehabilitation programs for lumbar surgery patients, three critical evidence gaps persist. First, the critical early recovery window—the first 3 months post-discharge, when patients transition from supervised in-hospital care to fully independent home-based self-management—has not been rigorously evaluated in a structured, protocol-driven manner. Most published trials either report outcomes only at discharge or lack comprehensive behavioral and functional assessments at the 3-month time point, which is a pivotal period for establishing rehabilitation habits and preventing early recurrence (11, 12). Second, there is substantial heterogeneity in mHealth application features across studies—ranging from simple text-message reminders to comprehensive platforms incorporating video instruction, AI-driven movement analysis, and risk stratification—making it difficult to identify which specific components drive observed improvements (13). Third, few studies have embedded theoretically grounded behavior change frameworks into their intervention design or used advanced statistical methods (e.g., regression modeling controlling for confounders) to isolate the independent contribution of the digital intervention from other concurrent care components. The present study addresses these gaps by evaluating a comprehensive mHealth platform with AI-powered movement monitoring, behavior change support, and risk stratification during the 3-month postoperative period, with regression analyses to quantify the independent effect of the intervention. We acknowledge that longer-term outcomes (>6 months) warrant separate investigation in future studies.

In recent years, mobile internet technology has gained momentum in healthcare and nursing, significantly enhancing both the quality and efficiency of patient follow-up care. A landmark multicenter prospective randomized controlled trial examined the efficacy and safety of mobile phone-based rehabilitation programs for lumbar surgery patients. The results demonstrated that those using these mobile rehabilitation apps achieved marked improvements in functional recovery and pain management outcomes (14). However, despite the promising outcomes, there are several limitations in current research on mobile internet technology in postoperative care. First, many studies lack long-term follow-up data, making it difficult to assess the sustained effectiveness of mobile-based rehabilitation programs over time. Additionally, there is a significant variability in the design and implementation of mobile health (mHealth) apps, with differences in the features offered, such as real-time monitoring, patient education, and communication with healthcare providers. This variability can affect the generalizability of findings across different patient populations and healthcare settings.

Therefore, this paper will discuss the application effect of mobile Internet technology in the postoperative follow-up management of patients with lumbar disc herniation, in order to provide an empirical basis for the postoperative rehabilitation of such patients.

## 2 Objects and methods

### 2.1 Study design

This was a randomized controlled study involving patients diagnosed with lumbar disc herniation (LDH) who underwent percutaneous discectomy at the Fourth Affiliated Hospital of Zhejiang University School of Medicine between January 2024 and October 2024. The study protocol was approved by the Hospital's Medical Ethics Committee (Approval No: (2021) Lun Shen Zi (ZJ-4H1011)). All participants and their families were fully informed about the study and provided written informed consent.

Randomization was performed using a computer-generated random number sequence with a 1:1 allocation ratio, stratified by herniated level (L3–L4, L4–L5, L5–S1) using permuted blocks of sizes 4 and 6 to ensure balance within strata. The randomization sequence was generated independently by a biostatistician not involved in participant recruitment or outcome assessment. Allocation concealment was achieved using sequentially numbered, opaque, sealed envelopes. After eligibility confirmation and baseline assessment, a research nurse who was not involved in outcome assessment opened the sequentially numbered envelopes in strict numerical order and assigned participants to the corresponding groups. To maintain allocation concealment, the sequence was not revealed to any study personnel involved in recruitment or baseline data collection until the moment of assignment. Due to the nature of the intervention, participants and healthcare providers delivering the intervention could not be blinded to group assignment (performance bias). However, outcome assessors—including the research nurse administering all scales and the radiologist evaluating recurrence—were blinded to group allocation. Blinding integrity was maintained by ensuring that assessors did not have access to participants' mini-program usage data or follow-up records.

Inclusion criteria were as follows: (1) diagnosis of LDH confirmed by physical and imaging examinations; (2) presence of severe nerve compression symptoms with no improvement after 6 months of conservative treatment; (3) successful completion of percutaneous discectomy; (4) age between 18 and 59 years; (5) conscious, with good cardiopulmonary function and no surgical contraindications; (6) education level of junior high school or above, with adequate reading ability; (7) possession and ability to use a smartphone.

Exclusion criteria included: (1) history of prior lumbar spine surgery; (2) other spinal conditions (e.g., spinal tuberculosis); (3) severe lumbar instability; (4) other serious complications; (5) illiteracy, cognitive impairment, or severe mental illness; (6) withdrawal from the study for any reason.

### 2.2 Sample size estimation

The sample size was calculated using the formula for comparing two means: *n* = 2 × [(*uα* + *uβ*)/(*δ*/*σ*)]^2^ + 0.25 × *uα*^2^ (6). With a bilateral *α* = 0.05 (*uα* = 1.96), *β* = 0.1 (*uβ* = 1.28), and effect size *δ*/*σ* = 0.2, the required sample size per group was estimated to be approximately 43 (calculated using the formula *n* = 2[(*uα* + *uβ*)/(*δ*/*σ*)]^2^ + 0.25·*uα*^2^). To account for potential variability and to ensure adequate power, we targeted 50 per group. Accounting for an anticipated dropout rate of 10% (rather than 20%, as the follow-up period was short and previous studies in our center showed <5% loss at 3 months), a total of 102 patients were initially enrolled to ensure 100 completers. Patients were allocated to either the control group or the intervention group (51 each) strictly according to the computer-generated randomization sequence and sealed envelope procedure described in Section 2.1. No random number table was used. During follow-up, one patient from the intervention group withdrew due to relocation, and one from the control group was lost due to contact change and lack of family cooperation. Ultimately, 100 patients completed the study.

### 2.3 Intervention and quality control

The control group received routine follow-up management, which encompassed in-hospital postoperative health education, the establishment of discharge follow-up records, and guidance on postoperative functional exercises. From the first day after surgery until discharge, patients were instructed to actively perform straight-leg raising exercises, wear an abdominal binder, ambulate early, and practice ankle pumps to prevent deep vein thrombosis. Within the first week after surgery, patients were encouraged to gradually increase walking distance as tolerated. Beginning one week post-surgery, patients progressively advanced to the following core strengthening exercises: (1) five-point support: in a supine position with hips and knees flexed, feet hip-width apart, and upper arms on the mat, patients lifted their hips using foot, elbow, and shoulder support, held for 3–5 s, and then lowered slowly; this was repeated 20–30 times per set for 3 sets, with 1-min intervals. (2) Three-point support: after adapting to the five-point support, patients crossed their arms over the chest and supported themselves with both feet and shoulders. (3) Plank exercise: maintaining a straight body alignment with elbows under shoulders and feet on the ground, patients held the position for 30–60 s per set, completing 2–4 sets with 1-min rests. (4) Supine unilateral knee flexion: patients pulled one knee toward the chest, held for 10 s, and repeated on the opposite side. (5) Supine bilateral knee-to-chest: with hips and knees bent, patients held both knees and lifted them toward the chest for 10 s. (6) Standing lunge: with feet shoulder-width apart, patients stepped backward into a lunge to stretch the hip flexors, holding on each side. Telephone follow-ups were conducted monthly, and formal reviews were scheduled at 1 and 3 months after discharge.

Follow-up management based on mobile Internet technology was implemented as follows: The intervention group received follow-up management utilizing a mobile internet-based system. The first step involved establishing a follow-up management team, which consisted of one orthopedic physician, one rehabilitation physician, one software engineer, one head nurse (team leader), and four specialist nurses. All team members were thoroughly trained and qualified before assuming their roles. The team conducted a comprehensive review of relevant research related to follow-up management via mobile internet technology, utilizing databases such as Wanfang Data Resource System, CNKI, and PubMed. Based on this research and the specific needs of lumbar disc herniation surgery patients, a first draft of the follow-up protocol was developed (Table 1).

**Table 1:** Mobile internet-based follow-up management protocol.

Time point	Intervention content	Duration and frequency
At discharge	Nurses provided discharge guidance, distributed the mini-program QR code, and explained its use to help patients establish rehabilitation goals. Self-made health education prescriptions for lumbar disc herniation were provided to standardize daily behavior and diet. Patients were instructed to record daily plans and results, maintain a rehabilitation diary for self-monitoring, and check in on the mini-program platform to log training diaries and plan implementation records.	30 min/session
1 month post-discharge	Patients were educated that postoperative rehabilitation could be enhanced through behavioral change. They were instructed to perform daily rehabilitation exercises using the template provided in the Internet-based mini-program. The first telephone follow-up was conducted in the first week to assess mini-program usage, health status, and psychological state. Nurses supervised and encouraged functional exercise adherence and daily platform check-ins. Patients were reminded to sleep on a hard bed for 3 months post-surgery. Face-to-face communication was available for those with complications or discomfort.	Telephone follow-up: 10 min/session, once/week
2 months post-discharge	Team members conducted door-to-door visits to comprehensively assess the patient's condition face-to-face, inquire about uncomfortable symptoms, adjust functional exercise content based on recovery progress, identify areas needing improvement, and guide behavior feedback. Potential problems were predicted and resolved to reinforce patient confidence in recovery.	Telephone follow-up: 10 min/session, once every two weeks Door-to-door visit: 1 h/session, once
3 months post-discharge	Continued follow-up via telephone or WeChat to monitor the implementation of rehabilitation exercises. Communication with family members was encouraged to motivate patients to maintain adherence to the rehabilitation plan. The mini-program check-in was supervised. Based on the patient's progress and feedback, the rehabilitation plan was adjusted promptly to facilitate functional recovery.	Telephone follow-up: 10 min/session, once every two weeks

Technical architecture of the mini-program platform. The platform was developed as a WeChat mini-program (native Android/iOS cross-platform compatibility), running on a cloud-based infrastructure with a three-tier architecture: (1) front-end user interface (UI) layer, (2) application logic layer, and (3) back-end data management layer. The front-end UI was built using WeChat's native development framework (WXML + WXSS + JavaScript), which renders dynamically on users' smartphones. The application logic layer, hosted on Alibaba Cloud Elastic Compute Service (ECS), processes user requests, executes business rules (e.g., risk classification algorithms), and orchestrates data exchange between the front-end and back-end systems. The back-end data layer utilizes a MySQL relational database (version 8.0) to store structured patient data (demographics, assessment scores, recurrence events) and a Tencent Cloud Object Storage (COS) service for storing unstructured content (exercise video files, educational images, user-uploaded rehabilitation diaries). All data transmission between the user's device and the cloud server is encrypted using Transport Layer Security (TLS 1.3), and user authentication is managed via WeChat's OAuth 2.0 framework, ensuring compliance with China's Personal Information Protection Law.

The platform's AI-powered movement monitoring capability is implemented using a computer vision-based human pose estimation algorithm (OpenPose, version 1.7.0) adapted for mobile devices. When a patient performs an exercise (e.g., plank, five-point support), they position their smartphone camera at a distance of approximately 2–3 m. The system captures video frames at 15 frames per s, extracts 18 body keypoints (including shoulders, elbows, wrists, hips, knees, and ankles), and compares the detected joint angles against a pre-defined template for the prescribed exercise. Deviations exceeding a pre-set threshold (e.g., >15 degrees for hip angle deviation in plank) trigger an on-screen corrective prompt (e.g., “Please keep your back straight and avoid excessive hip elevation”). The AI monitoring feature is a mandatory component for all patients in the intervention group—the system automatically initiates monitoring upon each exercise session. AI algorithm development and pre-launch validation. The AI-powered movement monitoring capability was developed and validated prior to clinical implementation through a three-phase process. Phase 1: Algorithm development and training. The pose estimation algorithm (OpenPose, version 1.7.0) was configured to extract 18 body keypoints from video frames captured at 15 frames per second. A training dataset of 2,500 annotated exercise videos was collected from 50 healthy volunteers (25 males, 25 females; age range 22–55 years) performing the six prescribed exercises (five-point support, three-point support, plank, supine unilateral knee flexion, supine bilateral knee-to-chest, and standing lunge) under standardized recording conditions (smartphone camera distance 2.5–3.0 m, ambient lighting 300–500 lux). Each video was annotated by two independent physical therapists who classified each exercise repetition as “correct form” or “form error” based on predefined joint angle criteria (e.g., hip angle deviation >15 from neutral for plank; knee extension <160 for standing lunge). Inter-annotator agreement was high (*κ* = 0.89, 95% CI: 0.84–0.93). The algorithm's joint angle detection accuracy was benchmarked against a Vicon motion capture system in a subset of 100 videos, yielding a mean absolute error of 3.2 ± 1.8 for hip angle and 2.8 ± 1.5 for knee angle. Phase 2: Validation dataset and reference standard. A separate validation dataset of 500 videos (100 videos per exercise type) was collected from an additional 20 healthy volunteers who were not involved in the training phase. These videos were independently evaluated by three rehabilitation physicians (with ≥5 years of experience in spinal rehabilitation) who served as the reference standard. Each physician independently reviewed all 500 videos and classified exercise performance as “correct” (all joint angles within predefined thresholds) or “error” (any joint angle deviation exceeding thresholds). Final reference standard classification was determined by majority consensus (≥2 of 3 physicians). Physicians were blinded to the algorithm's classifications. Phase 3: Validation results. The algorithm's performance was evaluated against the physician consensus reference standard. For detecting exercise form errors, the system achieved a sensitivity of 85.6% (95% CI: 82.1%–88.7%), specificity of 89.2% (95% CI: 86.0%–92.1%), positive predictive value of 86.3% (95% CI: 82.8%–89.4%), and negative predictive value of 88.6% (95% CI: 85.3%–91.5%), with an overall accuracy of 87.3% (95% CI: 84.9%–89.5%). The agreement between the algorithm and physician consensus was substantial (Cohen's *κ* = 0.74, 95% CI: 0.68–0.80). Supplementary Table S2 presents the confusion matrix and performance metrics stratified by exercise type. Performance varied modestly across exercise types: the highest accuracy was observed for plank (91.2%) and five-point support (89.6%), while standing lunge showed the lowest accuracy (83.4%), primarily due to challenges in detecting subtle knee flexion angle deviations in the sagittal plane. These validation results were considered acceptable for clinical deployment, with the rehabilitation physician serving as the final arbiter for any ambiguous cases flagged by the system.

The platform includes the following functional modules: (1) Daily Reminders (mandatory)—push notifications at 8 a.m. and 2 p.m. prompting patients to perform functional exercises; adherence is tracked via the daily check-in function. (2) Functional Exercise (mandatory)—video-based exercise instructions, including methods, steps, and precautions; the AI movement monitoring system is triggered automatically upon the patient's initiation of an exercise session, providing real-time posture feedback. (3) Health Education (optional but encouraged)—regular rehabilitation education and health knowledge content on lifestyle adjustments after lumbar disc herniation surgery; patients are not required to view all materials, but usage is logged. (4) Doctor–Patient Interaction (optional)—patients can communicate with designated doctors daily via text or voice messages; usage is quantified by number of messages exchanged per week. (5) Sharing Area (optional)—patients can share experiences based on their disease and cultural backgrounds. (6) Daily Check-in (mandatory)—patients upload progress reports (text and optionally video) on their exercises; the check-in data enables nurses to adjust exercise prescriptions and send remote reminders if exercises are missed. Check-in adherence is measured as the percentage of days with completed check-ins divided by total expected check-in days over the follow-up period. (7) Smart Questionnaire (mandatory at scheduled time points)—facilitates data collection through patient surveys at discharge, 1 month, and 3 months. (8) One-click Help (mandatory, always available)—provides 24/7 access to on-duty doctors for immediate assistance and guidance for patients experiencing sudden pain or muscle weakness.

Following this, an “Internet + mini program” platform was created. The follow-up management team collaborated with software engineers from the information department to design a comprehensive, interactive platform. The information department was responsible for software debugging, operational tasks, and ongoing system maintenance. The mini-program platform includes several functional modules to facilitate patient engagement and management. These modules are: Daily Reminders, which push notifications to remind patients to perform functional exercises at 8 a.m. and 2 p.m.; Functional Exercise, which provides exercise instructions via video, including methods, steps, and precautions; Health Education, offering regular rehabilitation education and health knowledge on topics such as lifestyle adjustments after lumbar disc herniation surgery; Doctor–Patient Interaction, where patients can communicate with designated doctors daily; a Sharing Area, allowing patients to share experiences based on their disease and cultural backgrounds; Daily Check-in, where patients upload progress reports on their exercises, enabling nurses to adjust exercise prescriptions and send remote reminders if exercises are missed; and a Smart Questionnaire, which facilitates data collection through patient surveys.

To quantify patient engagement with the digital intervention, the following adherence metrics were recorded for each participant in the intervention group over the 3-month follow-up period (see Supplementary Table S1 for module-level details): (1) daily check-in completion rate (number of days with completed check-in/total days in the active follow-up period × 100%); (2) functional exercise video viewing count (total number of video plays); (3) AI movement monitoring usage frequency (number of exercise sessions with AI monitoring activated); (4) doctor–patient interaction frequency (number of messages exchanged per week); and (5) health education material access frequency (number of materials opened). These metrics were summarized weekly by the data manager and reviewed by the head nurse, who provided targeted encouragement to patients with low engagement (e.g., check-in rate < 70% for two consecutive weeks triggered a follow-up phone call from the specialist nurse to identify barriers and provide additional support).

To ensure the quality of follow-up care, several measures were implemented. The Hierarchical Safety Assessment System involved evaluating patients' risk levels based on their surgical methods, age, and comorbidities. Patients were categorized into red, yellow, and green risk levels, with tailored follow-up plans for each category. High-risk patients (red label) received weekly home visits, medium-risk patients (yellow label) were checked via video calls twice a week, and low-risk patients (green label) were monitored through the platform's AI system. The Cognitive Difference Intervention was implemented during the first follow-up visit to assess patients' understanding of key rehabilitation points, such as pain tolerance and emergency management. Patients with scores below 80 points received additional health education until their score exceeded 80. Additionally, the platform automatically generated an electronic version of the “Informed Consent Form for Home Rehabilitation,” which outlined exercise intensity thresholds and contraindications, requiring patients to electronically confirm their understanding by signing. A two-person verification system was established, where the head nurse reviewed the exercise logs of high-risk patients weekly, and rehabilitation physicians responded to any early warning signs, such as persistent pain, within 48 h. Furthermore, a prominent “one-click help” button was integrated into the platform, providing 24/7 access to on-duty doctors for immediate assistance and guidance for patients experiencing sudden pain or muscle weakness.

To ensure transparency regarding intervention delivery and to enable meaningful interpretation of engagement data, each module was prospectively classified as either “mandatory” or “optional” based on its role in the core intervention protocol (see Supplementary Table S1 for detailed operational definitions). Mandatory modules (Daily Reminders, Functional Exercise, Daily Check-in, Smart Questionnaire, and One-click Help) were automatically activated for all patients in the intervention group, and non-use constituted protocol deviation. These modules delivered the essential components of the intervention: exercise prescription, posture monitoring, symptom surveillance, and emergency access. Optional modules (Health Education, Doctor–Patient Interaction, and Sharing Area) were available for patient-initiated use; non-use did not constitute protocol deviation. Usage of optional modules was logged to describe patient preferences and supplemental engagement patterns, but adherence analyses focused primarily on mandatory module completion rates to assess intervention fidelity. This classification framework ensured that patients received a standardized core intervention while allowing flexibility for supplementary features based on individual needs and preferences.

### 2.4 Data collection

Data were collected at three time points: upon discharge, 1 month after discharge, and 3 months after discharge. The same researcher administered all assessments using consistent measurement tools to evaluate patients' self-management ability, exercise compliance, and lumbar spine function. In addition, rehabilitation outcomes and recurrence rates were assessed 3 months after discharge.

Self-management ability was measured using the Adult Health Self-Management Skill Rating Scale (AHSMSRS) (15). This scale comprises three subscales and 38 items, each rated on a 5-point Likert scale. The subscales include self-management behavior (14 items, total score 70), self-management environment (10 items, total score 50), and self-management cognition (14 items, total score 70). Higher scores indicate better self-management ability.

Exercise compliance was evaluated with the Functional Exercise Adherence Scale for Orthopedic Patients (16), which consists of 15 items across three dimensions: physical, psychological, and active learning-related adherence. Each item is scored from 1 to 5 using a Likert scale, yielding a total score between 15 and 75. Scores below 20 indicate low adherence, scores between 20 and 55 indicate partial adherence, and scores of 55 or higher indicate high adherence. The total score is positively correlated with exercise compliance.

Lumbar spine function was assessed using the Japanese Orthopaedic Association (JOA) Lumbar Spine Function Scale (17), which has demonstrated reliability of 0.783 and validity of 0.842. The JOA scale includes four domains: subjective symptoms (9 points), clinical signs (6 points), limitations in daily activities (14 points), and bladder function (ranging from −6 to 0 points). The total score is 29 points, with lower scores indicating more severe functional impairment.

Rehabilitation effects was evaluated according to the “Guidelines for the Diagnosis, Treatment and Rehabilitation Management of Lumbar Disc Herniation” (18). Outcomes were classified as excellent (no residual low back or leg pain, no movement restrictions, normal work and daily life), good (occasional low back or leg pain, basically restored motor function, ability to perform light work), fair (significant low back or leg pain affecting life and work), or poor (severe low back or leg pain preventing return to original job). The excellent rate was calculated as the percentage of patients with excellent or good outcomes. Recurrence was defined as re-protrusion at the same segment and side within 3 months postoperatively, accompanied by radicular symptoms such as radiating pain or numbness, or new symptoms with imaging confirmation of disc herniation. Contralateral protrusion and adjacent segment degeneration were excluded. Recurrence rate was calculated as the number of recurrent cases divided by the total number of cases, multiplied by 100%.

### 2.5 Statistical methods

Statistical analyses were performed using SPSS version 22.0. Normally distributed continuous data were presented as mean ± standard deviation. The Shapiro–Wilk test was used to assess normality for continuous outcome variables (self-management ability, rehabilitation exercise adherence, and JOA scores) at each time point. All primary outcome variables were found to meet the normality assumption (*P* > 0.05 for all), supporting the use of parametric tests (repeated-measures ANOVA) for these measures. For baseline descriptive data, age, disease duration, and BMI were also normally distributed and are presented as mean ± standard deviation; however, operation time and hospital stay were not normally distributed and are therefore presented as median (interquartile range) and compared using the Mann–Whitney *U* test. Mauchly's test of sphericity was performed for repeated-measures ANOVA; when the sphericity assumption was violated (*P* < 0.05), the Greenhouse-Geisser correction was applied. Repeated-measures analysis of variance (ANOVA) was employed to compare self-management ability, rehabilitation exercise adherence scores, and JOA scores between the two groups over time. Within-group comparisons across different time points were conducted using the Bonferroni *post-hoc* test. Continuous variables that did not follow a normal distribution were expressed as median (interquartile range) and compared between groups using the Mann–Whitney *U* test. Categorical data were compared using the chi-square test when all expected cell frequencies were ≥5; when expected frequencies were <5, Fisher's exact test was used instead, while ordinal data were analyzed with the Wilcoxon rank-sum test. All analyses were conducted on a per-protocol basis, as only two participants were lost to follow-up (2.0%); To assess the robustness of our findings, we performed a *post-hoc* sensitivity analysis using multiple imputation with fully conditional specification (FCS) for the two missing cases at 3 months; the results of this sensitivity analysis are reported in Section 3.9. To identify independent factors associated with excellent rehabilitation outcome (excellent/good vs. fair/poor), binary logistic regression analysis was performed, with results expressed as adjusted odds ratios (ORs) and their 95% confidence intervals (CIs). Multiple linear regression analyses were conducted to assess the independent influences of the intervention and other baseline factors on the primary continuous outcome measures (3-month self-management behavior score, exercise adherence score, and JOA score), with results reported as unstandardized (B) and standardized (*β*) coefficients. Model fit was assessed using the Nagelkerke *R*^2^ for logistic regression and adjusted *R*^2^ for linear regression. A two-sided *P* < 0.05 was considered statistically significant.

## 3 Results

### 3.1 Patient enrollment and baseline characteristics

A total of 102 patients who underwent percutaneous discectomy for lumbar disc herniation were initially enrolled in this study. During the follow-up period, two patients were lost: one in the intervention group due to relocation and one in the control group due to loss of contact. Ultimately, 100 patients completed the study, with 50 in each group. No significant differences were observed in baseline demographic and clinical characteristics between the two groups (all *P* > 0.05), indicating successful randomization. The participant flow and baseline comparisons are summarized in Table 2.

**Table 2:** Baseline characteristics of participants.

Characteristic	Control Group (*n* = 50)	Intervention Group (*n* = 50)	Statistics	*P*-value
Gender, *n* (%)			*χ*^2^ = 0.167	0.683
Male	31 (62.0)	29 (58.0)		
Female	19 (38.0)	21 (42.0)		
Age (years)	43.34 ± 5.75	43.18 ± 5.27	*t* = 0.145	0.885
Disease duration (months)	14.64 ± 2.47	14.78 ± 2.32	*t* = 0.292	0.771
BMI (kg/m^2^)	24.17 ± 2.52	24.68 ± 2.45	*t* = 1.026	0.307
Herniated level, *n* (%)			*χ*^2^ = 0.179	0.914
L3–L4	7 (14.0)	8 (16.0)		
L4–L5	24 (48.0)	22 (44.0)		
L5–S1	19 (38.0)	20 (40.0)		
Operation time (min)	48.0 (40.0–57.0)	47.0 (38.0–58.0)	*Z* = 0.538	0.592
Hospital stay (days)	3.0 (2.0–4.0)	3.0 (2.0–5.0)	*Z* = 0.251	0.802

BMI, body mass index. Data are presented as mean ± standard deviation, *n* (%), or median (interquartile range).

### 3.2 Comparison of self-management ability between groups

Repeated-measures ANOVA revealed that the main effects of time and group, as well as the time × group interaction effect on self-management ability scores, were all statistically significant (all *P* < 0.001). The scores for self-management behavior, self-management environment, and self-management cognition at discharge, 1 month, and 3 months post-discharge are presented in Table 3. The intervention group demonstrated greater improvement in all three subscales over time compared to the control group.

**Table 3:** Comparison of self-management ability scores between the two groups (mean ± SD).

Group	n	At discharge	1 month post-discharge	3 months post-discharge	Fgroup (*P*)	Ftime (*P*)	Finteraction (*P*)
Self-management behavior							
Control	50	38.53 ± 3.44	44.28 ± 5.73	52.62 ± 5.87	16.452 (<0.001)	14.626 (<0.001)	14.276 (<0.001)
Intervention	50	38.46 ± 3.67	49.35 ± 5.46	60.55 ± 5.42			
Self-management environment							
Control	50	28.53 ± 3.74	32.25 ± 4.63	35.64 ± 5.38	10.346 (<0.001)	11.482 (<0.001)	17.648 (<0.001)
Intervention	50	28.27 ± 3.66	34.45 ± 5.34	41.72 ± 6.23			
Self-management cognition							
Control	50	40.64 ± 3.37	45.35 ± 4.28	52.37 ± 5.92	13.185 (<0.001)	16.448 (<0.001)	13.722 (<0.001)
Intervention	50	40.32 ± 3.42	48.41 ± 5.23	61.48 ± 6.25			

SD, standard deviation.

### 3.3 Comparison of rehabilitation exercise adherence between groups

Repeated-measures ANOVA indicated that the main effects of time and group, as well as the time × group interaction effect on rehabilitation exercise adherence, were all statistically significant (all *P* < 0.001). The adherence scores for both groups at discharge, 1 month, and 3 months post-discharge are presented in Table 4. The intervention group maintained significantly higher exercise adherence over time compared to the control group.

**Table 4:** Comparison of rehabilitation exercise adherence between the two groups (mean ± SD).

Group	*n*	At discharge	1 month post-discharge	3 months post-discharge	Fgroup (*P*)	Ftime (*P*)	Finteraction (*P*)
Control	50	56.38 ± 5.64	47.54 ± 5.33	40.53 ± 4.83	17.625 (<0.001)	13.413 (<0.001)	10.132 (<0.001)
Intervention	50	57.46 ± 6.12	54.73 ± 6.64	53.67 ± 6.42			

According to the scale criteria, scores ≥55 indicate high adherence, scores between 20 and 55 indicate partial adherence. The intervention group's mean score of 53.67 at 3 months is therefore classified as partial adherence, although it remained significantly higher than the control group's score of 40.53.

### 3.4 Comparison of lumbar spine function (JOA scores) between groups

Repeated-measures ANOVA revealed that the main effects of time and group, as well as the time × group interaction effect on JOA scores, were all statistically significant (all *P* < 0.001). The JOA scores for both groups at discharge, 1 month, and 3 months post-discharge are presented in Table 5. The intervention group showed significantly greater improvement in lumbar spine function over time compared to the control group.

**Table 5:** Comparison of JOA scores between the two groups (mean ± SD).

Group	*n*	At discharge	1 month post-discharge	3 months post-discharge	Fgroup (*P*)	Ftime (*P*)	Finteraction (*P*)
Control	50	13.45 ± 1.27	15.27 ± 2.21	19.45 ± 2.74	19.356 (<0.001)	6.489 (<0.001)	13.543 (<0.001)
Intervention	50	13.28 ± 1.24	18.53 ± 2.36	23.26 ± 3.48			

SD, standard deviation; JOA, Japanese Orthopaedic Association.

### 3.5 Comparison of excellent rehabilitation rate and recurrence rate between groups

At 3 months post-discharge, the excellent rehabilitation rate was significantly higher in the intervention group (92.00%) than in the control group (76.00%) (*χ*^2^ = 4.762, *P* = 0.029). The recurrence rate was 0.00% (0/50) in the intervention group compared to 10.00% (5/50) in the control group; however, due to the low expected cell frequency (2.5) in the 2 × 2 table, Fisher's exact test was applied and yielded a two-sided *P* value of 0.056, indicating a trend toward reduced short-term recurrence that did not reach the conventional threshold for statistical significance (Table 6). This finding should be interpreted with caution, as the study was not powered to detect differences in recurrence rates. The absence of events in the intervention group precludes a definitive conclusion, and the observed 10-percentage-point difference, while clinically meaningful, requires confirmation in larger, adequately powered studies.

**Table 6:** Comparison of excellent rehabilitation rate and recurrence rate between the two groups [*n* (%)].

Group	*n*	Excellent	Good	Fair	Poor	Excellent Rate	Recurrence Rate
Control	50	20 (40.0)	18 (36.0)	7 (14.0)	5 (10.0)	38 (76.0)	5 (10.0)
Intervention	50	31 (62.0)	15 (30.0)	4 (8.0)	0 (0.0)	46 (92.0)	0 (0.0)
Statistics						*χ*^2^ = 4.762	Fisher's exact test
*P*-value						0.029	0.056

Excellent rate = (Excellent + Good) cases/total cases × 100%. For recurrence rate comparison, Fisher's exact test was used because the expected frequency in one cell (intervention group with recurrence) was 2.5, which is below the threshold of 5 for valid *χ*^2^ approximation. The two-sided *P* value of 0.056 indicates a trend toward lower recurrence in the intervention group that does not reach conventional statistical significance. The excellent rehabilitation rate remained significantly higher in the intervention group (*χ*^2^ = 4.762, *P* = 0.029).

### 3.6 Logistic regression analysis of factors associated with excellent rehabilitation

To further identify factors independently associated with achieving an excellent rehabilitation outcome, a binary logistic regression analysis was performed. The dependent variable was defined as excellent rehabilitation (yes/no), based on the outcomes classified in Section 3.5. Independent variables included group assignment (intervention vs. control), age, gender, disease duration, body mass index (BMI), herniation level, and operation time.

The logistic regression model was statistically significant (*χ*^2^ = 28.74, *P* < 0.001), explaining 42.3% (Nagelkerke *R*^2^) of the variance in excellent rehabilitation outcome and correctly classifying 88.0% of cases. As shown in Table 7, participation in the mobile Internet-based follow-up management program was the strongest independent predictor of achieving an excellent outcome. Patients in the intervention group had over eight times higher odds of excellent recovery compared to those in the control group (adjusted OR = 8.42, 95% CI: 2.75–25.78, *P* < 0.001). Furthermore, a shorter disease duration was also significantly associated with a higher likelihood of excellent recovery (adjusted OR = 0.86, 95% CI: 0.75–0.98, *P* = 0.026). No other included variables demonstrated a statistically significant independent association with the outcome.

**Table 7:** Logistic regression analysis of factors associated with excellent rehabilitation outcome.

Variable	*Β*	SE	Wald	*P*-value	Adjusted OR	95% CI for OR
Group (Intervention)	2.13	0.55	15.12	<0.001	8.42	2.75–25.78
Age (years)	−0.04	0.04	0.83	0.362	0.96	0.88–1.05
Gender (Male)	0.32	0.53	0.37	0.545	1.38	0.48–3.93
Disease duration (months)	−0.15	0.07	4.95	0.026	0.86	0.75–0.98
BMI (kg/m^2^)	−0.08	0.10	0.66	0.418	0.92	0.76–1.12
Herniation level (L4-L5)^1^	0.45	0.59	0.59	0.441	1.57	0.50–4.98
Operation time (min)	−0.02	0.02	0.72	0.397	0.98	0.94–1.03
Constant	1.98	1.86	1.13	0.287	7.24	

OR, odds ratio; CI, confidence interval; SE, standard error. The model is adjusted for all variables listed in the table. Reference categories: Group = Control; Gender = Female; Herniation level^1^ = L3–L4/L5–S1 (combined due to smaller sample sizes). A significant *P*-value (<0.05) indicates an independent association with the excellent rehabilitation outcome.

### 3.7 Linear regression analysis of factors influencing key outcome scores

To quantify the independent influence of various factors, including the intervention, on the continuous primary outcomes, multiple linear regression analyses were conducted. The dependent variables were the 3-month post-discharge scores for self-management behavior, rehabilitation exercise adherence, and JOA score. Independent variables included group assignment, age, gender, disease duration, and baseline score at discharge.

The regression model for self-management behavior was significant [*F*(5, 94) = 32.15, *P* < 0.001, Adjusted *R*^2^ = 0.612]. As shown in Table 8, participation in the intervention group was the strongest positive predictor of a higher 3-month self-management behavior score (*β* = 0.712, *P* < 0.001), after controlling for other variables. A higher baseline score at discharge was also a significant positive predictor (*β* = 0.205, *P* = 0.002). No other variables were significantly associated with the outcome.

**Table 8:** Multiple linear regression analysis of factors associated with 3-month outcome scores.

Dependent variable/predictor	*B*	SE	*β*	*t*-value	*P*-value	95% CI for *B*
Self-management behavior						
(Constant)	15.24	3.58		4.26	<0.001*	8.13–22.35
Group (intervention)	7.93	0.82	0.712	9.67	<0.001*	6.30–9.56
Baseline Score	0.41	0.13	0.205	3.15	0.002*	0.15–0.67
Age (years)	−0.05	0.06	−0.054	−0.83	0.407	−0.17 to 0.07
Gender (Male)	0.61	0.78	0.051	0.78	0.436	−0.94 to 2.16
Disease duration (months)	−0.11	0.14	−0.049	−0.79	0.432	−0.39 to 0.17
Rehabilitation adherence						
(Constant)	12.88	3.82		3.37	0.001*	5.29–20.47
Group (Intervention)	13.14	1.41	0.694	9.32	<0.001*	10.34–15.94
Baseline score	0.38	0.11	0.223	3.45	0.001*	0.16–0.60
Age (years)	−0.08	0.07	−0.077	−1.14	0.257	−0.22 to 0.06
Gender (Male)	0.87	0.94	0.061	0.93	0.356	−0.99 to 2.73
Disease duration (months)	−0.31	0.16	−0.128	−1.97	0.051	−0.62 to 0.00
JOA score						
(Constant)	10.35	2.98		3.47	0.001*	4.43–16.27
Group (intervention)	3.81	0.52	0.654	7.33	<0.001*	2.78–4.84
Baseline Score	0.59	0.12	0.287	4.92	<0.001*	0.35–0.83
Age (years)	−0.09	0.04	−0.132	−2.03	0.045*	−0.17 to −0.01
Gender (Male)	0.55	0.57	0.067	0.96	0.338	−0.59 to 1.69
Disease Duration (months)	−0.12	0.09	−0.085	−1.31	0.192	−0.30 to 0.06

*P* < 0.05 (statistically significant). All significant *P*-values are also shown in bold for emphasis. Group coding: 0 = Control, 1 = Intervention; Gender: 0 = Female, 1 = Male. *B* = unstandardized coefficient; SE = standard error; *β* = standardized coefficient; CI = confidence interval. Baseline score refers to the respective score measured at discharge.

The model for exercise adherence was also significant [*F*(5, 94) = 29.84, *P* < 0.001, Adjusted *R*^2^ = 0.589]. Group assignment was again the strongest significant predictor (*β* = 0.694, *P* < 0.001), with the intervention group associated with markedly higher adherence scores. A higher baseline adherence score was also a significant positive predictor (*β* = 0.223, *P* = 0.001). Shorter disease duration showed a borderline significant positive association with better adherence (*β* = −0.128, *P* = 0.051).

The regression model for JOA score was significant [*F*(5, 94) = 38.22, *P* < 0.001, Adjusted *R*^2^ = 0.651]. Similar to the other models, being in the intervention group was the strongest independent factor associated with a superior JOA score (*β* = 0.654, *P* < 0.001). The baseline JOA score at discharge was a significant positive predictor (*β* = 0.287, *P* < 0.001). Older age was a weak but significant negative predictor of JOA score (*β* = −0.132, *P* = 0.045).

### 3.8 Patient engagement with the digital intervention

To assess the fidelity of the mobile Internet-based intervention, we prospectively tracked patient engagement across all eight functional modules of the mini-program platform. Per the protocol defined in Section 2.3 and Supplementary Table S1, modules were classified as “mandatory” (Daily Reminders, Functional Exercise, Daily Check-in, Smart Questionnaire, and One-click Help) or “optional but encouraged” (Health Education, Doctor–Patient Interaction, and Sharing Area). Mandatory modules were automatically activated for all patients in the intervention group and required for protocol adherence; optional modules were available for patient-initiated use, with usage logged for descriptive reporting. The following adherence metrics were recorded for the 50 patients in the intervention group over the 3-month follow-up period (Supplementary Table S2).

For mandatory modules: the mean daily check-in completion rate (defined as the percentage of days with completed check-ins relative to total expected check-in days over the 3-month period) was 84.6% (SD = 11.2%); the mean functional exercise video viewing count was 22.4 (SD = 8.7) plays per patient; the mean AI movement monitoring usage frequency was 16.3 (SD = 6.1) sessions per patient (representing 85.2% of all logged exercise sessions, indicating that patients predominantly used the AI-monitored mode rather than unsupervised exercise); and the Smart Questionnaire completion rate was 100% at 1 month and 98% at 3 months (one patient delayed completion by 3 days due to travel). The One-click Help function was activated by 6 patients (12.0%) on a total of 9 occasions, all of which were resolved within 24 h by the on-duty physician, and none required emergency department referral.

For optional modules: the mean doctor–patient interaction frequency was 2.1 (SD = 1.4) messages exchanged per week, with substantial between-patient variability (range: 0.3–6.8 messages/week); the mean health education material access frequency was 12.5 (SD = 5.3) materials opened per patient, with 42 patients (84.0%) accessing at least one material per week during the first month, declining to 28 patients (56.0%) by the third month; and the Sharing Area was used by 23 patients (46.0%), with a total of 67 posts generated over the follow-up period. The observed variability in optional module usage, combined with the high adherence to mandatory modules, suggests that the core intervention components were delivered as intended, while allowing for patient-preference flexibility in supplementary features.

These engagement data confirm that the digital intervention was implemented with high fidelity for mandatory components, with adherence rates exceeding 80% for core protocol elements (daily check-in and AI-monitored exercise). Optional module usage showed expected variability, reflecting patient preferences without compromising the delivery of essential intervention components. The pattern of sustained check-in adherence (month-by-month: 87.2% in month 1, 84.1% in month 2, 82.5% in month 3) indicates that patient engagement remained stable over the 3-month follow-up period, with only a modest 4.7% decline between the first and third months.

The AI movement monitoring system deployed in this study had been pre-validated against a physician consensus reference standard through a three-phase process (training on 2,500 videos, validation on 500 independent videos), with overall sensitivity of 85.6%, specificity of 89.2%, and substantial inter-rater agreement (*κ* = 0.74) (Supplementary Table S3). For the 50 patients in the intervention group, the following adherence metrics were recorded over the 3-month follow-up period: the mean daily check-in completion rate was 84.6% (SD = 11.2%), the mean functional exercise video viewing count was 22.4 (SD = 8.7) plays per patient, the mean AI movement monitoring usage frequency was 16.3 (SD = 6.1) sessions per patient, the mean doctor–patient interaction frequency was 2.1 (SD = 1.4) messages per week, and the mean health education material access frequency was 12.5 (SD = 5.3) materials opened per patient. These data indicate that the intervention group actively engaged with the prescribed digital components throughout the follow-up period.

### 3.9 Sensitivity analysis for missing data

To assess the robustness of our findings to the loss of two participants during follow-up (one in each group), we performed a sensitivity analysis using multiple imputation with fully conditional specification (FCS) to impute missing outcome data at 3 months post-discharge. The imputation model included all baseline covariates (age, gender, disease duration, BMI, herniation level, operation time) and all available outcome measurements at discharge and 1 month. Five imputed datasets were generated, and results were pooled using Rubin's rules. The intervention effect remained statistically significant for all primary outcomes in the imputed analyses: self-management behavior (*β* = 0.698, 95% CI: 0.612–0.784, *P* < 0.001), rehabilitation exercise adherence (*β* = 0.681, 95% CI: 0.593–0.769, *P* < 0.001), and JOA score (*β* = 0.642, 95% CI: 0.556–0.728, *P* < 0.001). These results were consistent with the per-protocol analyses reported in Section 3.7, confirming that the minimal loss to follow-up (2.0%) did not materially affect the study conclusions.

## 4 Discussion

The findings of this study demonstrate that follow-up management supported by mobile Internet technology significantly enhances self-management ability, lumbar spine function, rehabilitation exercise adherence, and clinical outcomes in patients undergoing surgery for LDH.

The repeated-measures analysis revealed statistically significant improvements in self-management ability within the intervention group, as reflected in the AHSMSRS scores. While percutaneous discectomy effectively relieves nerve root compression, successful long-term recovery depends on sustained rehabilitation and self-management (19). Conventional in-hospital care often falls short in meeting these continuous needs. The mobile Internet-based follow-up system addresses this gap through a structured “plan-execute-feedback” cycle facilitated by daily check-ins, AI-assisted movement monitoring, and nurse supervision. Multimedia education materials (videos and graphics) improved patient comprehension and retention of rehabilitation knowledge compared to conventional verbal instructions. Furthermore, electronic informed consent and risk-stratified management reduced errors due to poor health literacy, thereby significantly improving self-management behaviors and knowledge.

These findings are consistent with and extend the emerging evidence base for mHealth interventions in spinal rehabilitation. Hou et al. demonstrated in a multicenter randomized controlled trial that mobile phone-based rehabilitation programs produced significant improvements in functional recovery and pain management among lumbar surgery patients, with an effect size (Cohen's *d*) of approximately 0.65 for functional outcomes at 3 months (14). Our study similarly found a large effect size for JOA score improvement (Cohen's *d* = 0.89) at 3 months, suggesting that the addition of AI-powered movement monitoring and risk stratification may further enhance the efficacy of mobile-based approaches. Furthermore, our findings align with the theoretical framework proposed by Ridosh et al., who emphasized that self-management interventions should address not only behavioral components but also environmental and cognitive domains (15). The intervention in our study targeted all three domains—behavioral (daily check-in, video-guided exercises), environmental (sharing area, family involvement), and cognitive (health education, risk communication)—likely explaining the sustained improvements across all AHSMSRS subscales. Our study extends these findings by providing regression analyses that quantify the independent contribution of the digital intervention while controlling for potential confounders such as age, gender, disease duration, and baseline scores. However, we acknowledge that our study design does not allow us to isolate the specific effects of individual components (e.g., AI-powered monitoring vs. risk stratification vs. increased contact time), and the intervention should therefore be evaluated as a comprehensive package of care rather than as a technology-only solution.

The design principles and mechanisms identified in prior mHealth studies for spinal cord injury populations offer important insights for interpreting our results. One study on the development of a mobile application for self-management of spinal cord injury patients emphasized that successful mHealth interventions must be built upon a thorough understanding of patient-identified key requirements—including exercise monitoring, pain tracking, and peer support—rather than a top-down technology-driven approach (20). In our study, the requirements identification phase was conducted by a multidisciplinary team consisting of orthopedic physicians, rehabilitation physicians, and specialist nurses, who drew upon clinical experience, evidence-based guidelines, and the functional requirements documented in the mHealth literature. While the mini-program was not developed through a formal patient co-design process—a limitation we acknowledge—the functional modules (e.g., daily check-in, AI-powered movement monitoring, one-click help for sudden symptoms, and peer sharing area) were deliberately designed by the team to address the core patient-informed needs documented in prior studies, including exercise monitoring, pain/symptom tracking, and peer support. Future iterations should incorporate formal patient engagement in the requirements definition phase to strengthen user-centered design. This approach ensured that the mini-program addressed clinically meaningful and patient-centered needs, although future iterations should incorporate formal patient engagement in the requirements definition phase to strengthen user-centered design. Similarly, a Persian language self-management mobile application for individuals with spinal cord injury demonstrated that user engagement is critically dependent on features such as personalized feedback, progress visualization, and caregiver involvement (21). Our platform incorporated these mechanisms through AI-based posture correction (personalized feedback), graphical progress dashboards (progress visualization), and family member communication channels (caregiver involvement). The observation that our intervention group maintained high exercise adherence rates (mean 53.67 out of 75 at 3 months, compared to 40.53 in controls) suggests that these mechanisms effectively address the motivational and self-regulatory challenges that often undermine home-based rehabilitation adherence.

Significant improvements in JOA scores indicated better lumbar spine function among intervention group participants. This aligns with previous findings that mobile health technologies can enhance functional recovery in orthopedic patients (22). The intervention group adhered more closely to prescribed exercises through instructional videos, daily reminders, and check-ins. Real-time exercise monitoring with corrective feedback minimized incorrect postures—such as excessive hip flexion—which could cause secondary injury (23). Moreover, the combination of online supervision and risk-stratified follow-up—which included weekly telephone calls for all patients, bi-weekly or monthly calls depending on risk level, and targeted home visits for yellow- and red-risk patients—enabled continuous, personalized adjustment of exercise intensity, contributing to improved core muscle strength, stability, and functional capacity, particularly in daily activities such as standing and walking.

Rehabilitation exercise adherence was also significantly higher in the intervention group. This supports the results of Yuan et al., who reported that mobile “Internet + Medical” models increased exercise compliance in patients with non-specific low back pain (24). Traditional follow-up methods—such as telephone calls, SMS, and outpatient visits—often suffer from high loss-to-follow-up rates and fragmented data, impairing effective rehabilitation supervision. The mini-program's integrated approach, incorporating push notifications, clock-in features, video guidance, and family involvement, helped establish consistent exercise habits through principles of behavior change (25). It is worth noting that adherence may decline beyond three months; future iterations could incorporate gamified incentives—such as point-based rewards—to sustain long-term engagement.

However, it is important to interpret these positive findings with appropriate balance. The inability to blind participants and intervention providers represents a source of performance bias that may have inflated the observed effect sizes. Participants in the intervention group received substantially more healthcare contact (daily digital interactions, weekly telephone calls, and scheduled home visits) than the control group, raising the possibility that the “contact effect”—the increased attention and social support itself—contributed meaningfully to the superior outcomes observed, independent of the digital technology per se. In addition, the self-selection of patients willing to use smartphones and digital health technologies may have resulted in an inherently more motivated or health-literate sample, limiting the generalizability of the findings to less digitally engaged populations. Future studies employing a blinded, sham-controlled design (e.g., using a placebo mini-program without active AI monitoring) could better isolate the specific effects of the digital intervention components from the general benefits of increased attention.

Consistent with these functional and behavioral improvements, the intervention group exhibited a significantly higher excellent rehabilitation rate (92.00% vs. 76.00%) at three months post-discharge. The recurrence rate was 0.00% (0/50) in the intervention group compared to 10.00% (5/50) in the control group. However, as noted in Section 3.5, this difference did not reach statistical significance by Fisher's exact test (*P* = 0.056), likely due to the limited sample size and the low expected cell frequency. The observed 10-percentage-point difference is clinically meaningful but should be interpreted with caution; this study was not powered to detect differences in recurrence, and the non-significant result should not be interpreted as evidence of no effect. A larger sample is required for a definitive assessment.

These results are partially corroborated by studies in other surgical contexts, such as mobile Internet-based continuous care improving outcomes in breast cancer and hepatobiliary surgery patients (26, 27). Risk stratification (e.g., red-yellow-green classification) enabled early identification of high-risk patients, while standardized pain management protocols reduced exercise errors related to improper pain tolerance. By synergistically enhancing self-management, exercise compliance, and lumbar function, the mobile follow-up system effectively promotes short-term recovery. Whether these improvements translate into sustained reduction in late recurrence (beyond 6 months) requires longer-term follow-up, as acknowledged in the limitations section.

Additionally, we acknowledge that the per-protocol analysis may have introduced bias. However, given that only two participants were lost to follow-up (2.0%), and sensitivity analysis using multiple imputation for missing data yielded consistent results (data not shown), the impact on the overall conclusions is likely minimal.

Furthermore, the logistic and linear regression analyses robustly quantified the intervention's independent effect on patient outcomes. The mobile Internet-based management was identified as the strongest predictor for excellent recovery and significant improvements in self-management, adherence, and functional scores, even after adjusting for potential confounders such as age and disease duration. However, the effect size for JOA score improvement (Cohen's *d* = 0.89, calculated from the mean difference between groups at 3 months divided by the pooled standard deviation) was notably larger than the effect size (approximately 0.65) reported in a previous multicenter trial of mobile phone-based rehabilitation for lumbar surgery patients. While this difference may suggest that the addition of AI-powered movement monitoring and risk stratification could further enhance efficacy, we caution that the effect size estimate from our single-center study may be inflated due to performance bias and the increased contact time received by the intervention group. Furthermore, our study design does not allow us to isolate the specific effects of individual components (e.g., AI monitoring vs. risk stratification), and the intervention should therefore be evaluated as a comprehensive package of care rather than as a technology-only solution. The observed effect size should be interpreted with these limitations in mind. These statistical models not only reinforce the efficacy of the digital follow-up approach but also highlight its potential scalability and reproducibility. Future studies should focus on multi-center validation and longer follow-up periods to generalize these findings and further refine the intervention protocol through adaptive algorithms and personalized feedback mechanisms.

## 5 Limitations

Several limitations of this study should be acknowledged. First, the single-center design limits the generalizability of our findings to other healthcare settings with different patient populations, resource availability, and digital infrastructure. Multi-center studies involving diverse geographic and socioeconomic contexts are needed to validate the scalability of this intervention. Second, the follow-up period was limited to three months post-discharge. While this timeframe captures the early postoperative recovery phase and is consistent with previous studies reporting early recurrence rates following percutaneous discectomy (4, 5), it does not permit assessment of longer-term outcomes, including late recurrence (>6 months), sustained functional gains, or the durability of behavior change. The justification for selecting 3-month follow-up was based on: (1) clinical evidence indicating that the majority of early recurrences occur within the first 3 months post-surgery (28); (2) the acute rehabilitation window during which exercise adherence and self-management behaviors are most critical; and (3) logistical constraints related to patient retention beyond the immediate postoperative period. Nevertheless, future studies should extend follow-up to 12 months or longer to evaluate the long-term sustainability of the observed improvements. Third, the sample size (50 patients per group), while adequate to detect the pre-specified effect size (*δ*/*σ* = 0.2 with 90% power), may have limited the statistical power to detect smaller but clinically meaningful differences in subgroup analyses or to identify more complex interaction effects. The sample size was calculated based on the primary outcome (self-management ability score) and was not powered for secondary outcomes (e.g., recurrence rate), which explains both the wide confidence intervals observed for recurrence comparisons (0.00% vs. 10.00%) and the non-significant Fisher's exact test result (*P* = 0.056) despite a 10-percentage-point difference. With a zero event in the intervention group, the study had limited power to detect a statistically significant difference in recurrence, and the observed effect size should be interpreted with appropriate caution. The sample size calculation presented in Section 2.2 was based on the primary outcome of self-management ability. The study was not powered to detect differences in secondary outcomes such as recurrence rate, and the non-significant result for recurrence (*P* = 0.056) should be interpreted with this limitation in mind. A larger sample would be required to reliably estimate the recurrence-reduction effect and to conduct meaningful subgroup analyses. Future studies with larger sample sizes and longer follow-up periods are warranted to provide more precise estimates of recurrence reduction and to determine whether the observed trend represents a genuine protective effect. Fourth, the inclusion criteria restricted participation to patients aged 18–59 years with at least junior high school education and smartphone ownership. This selection criterion—necessary to ensure participants could read and operate the mini-program—introduces a significant selection bias and limits the applicability of our findings to older adults (≥60 years), who are at higher risk for LDH and postoperative complications, to individuals with lower educational attainment who may have limited health literacy, and to those without smartphones or digital literacy. The findings may not be generalizable to these vulnerable populations, which represent a substantial proportion of real-world LDH patients. Future research should explore simplified, low-technology adaptations (e.g., voice- or SMS-based interventions) tailored to older adults and digitally disadvantaged groups. Fifth, the per-protocol analytical approach (rather than intention-to-treat) may have introduced bias by excluding the two participants who were lost to follow-up. However, given the minimal loss rate (2.0%), this bias is likely small. The sensitivity analysis using multiple imputation for missing data, reported in Section 3.9, confirmed that the intervention effects remained statistically significant for all primary outcomes (*β* range: 0.642–0.698, all *P* < 0.001), supporting the robustness of our conclusions. Sixth, while the study employed rigorous methods to blind outcome assessors, the inability to blind participants and healthcare providers to group assignment represents an inherent limitation of behavioral intervention research and may have introduced performance bias. The differential attention received by intervention group participants (digital interactions, more frequent follow-up contacts) constitutes a “contact effect” that may have contributed to the observed effects independently of the technology itself. It is therefore not possible to attribute the superior outcomes exclusively to the digital components of the intervention, as the increased frequency and intensity of human interaction may have played a substantial role. Furthermore, the interpretation of the effect size is limited by the single-center design and the lack of a sham-controlled or attention-matched control group, which would be necessary to isolate the specific effects of the digital technology from the general benefits of increased attention. Seventh, the study did not systematically collect patient satisfaction or user experience data regarding the mini-program, nor did it evaluate the digital literacy levels of participants. Such data would provide valuable insights into the acceptability and usability of the platform and help identify barriers to engagement and areas for technical improvement. Future studies should incorporate validated user satisfaction and technology acceptance measures (e.g., the Technology Acceptance Model questionnaire) to inform iterative platform refinement and improve patient-centered design.

## 5 Conclusion

In conclusion, this study demonstrates that mobile Internet technology-based follow-up management significantly improves self-management capabilities, enhances lumbar function, and mitigates the decline in exercise adherence in patients after lumbar disc herniation surgery. Regression models confirm the intervention as a strong independent predictor of these superior outcomes. At 3 months post-discharge, the intervention group showed a trend toward lower recurrence rate (0.00% vs. 10.00%), but this difference did not reach statistical significance (Fisher's exact test, *P* = 0.056), and the study was not powered to detect recurrence differences. Longer-term follow-up in larger samples is required to determine whether the intervention confers a sustained reduction in recurrence risk. This approach provides a scalable, patient-centered model for extending structured rehabilitation beyond the hospital, offering a promising strategy for achieving short-term recovery and potentially improved long-term functional health, pending confirmation in future studies with extended follow-up durations. However, the findings should be interpreted with caution given the single-center design, the relatively small sample size, the inability to blind participants, and the potential for the observed effects to be partially driven by increased contact time rather than the digital intervention alone.

## Data Availability

The original contributions presented in the study are included in the article/Supplementary Material, further inquiries can be directed to the corresponding author.
