# Supplementary material for: The application effect of mobile internet technology in the postoperative follow-up management of patients with lumbar intervertebral disc protrusion
Source: Front Surg. 2026 Aug 31;13:1876447. doi: 10.3389/fsurg.2026.1876447 (PMC13572695; doi:10.3389/fsurg.2026.1876447)
Supplement: Supplementary file 1 [file Table1.docx]

**Table S1** Summary of mobile Internet-based mini-program functional modules: attributes, operationalization, and adherence metrics

| **Module name** | **Attribute** | **Operationalization** | **Quantitative adherence metric** |
| --- | --- | --- | --- |
| Daily Reminders | Mandatory | Push notifications at 8 a.m. and 2 p.m. prompting patients to perform functional exercises; adherence tracked via daily check-in function | Percentage of days with completed check-in / total expected check-in days × 100% (mean ± SD reported at 3 months) |
| Functional Exercise | Mandatory | Video-based exercise instructions including methods, steps, and precautions; AI-powered movement monitoring system (OpenPose, 18 keypoints, 15 fps) automatically triggered upon exercise initiation, providing real-time posture feedback with corrective prompts for angle deviations >15 | AI monitoring usage frequency: number of exercise sessions with AI monitoring activated (mean ± SD reported at 3 months); exercise video viewing count: total number of video plays per patient (mean ± SD) |
| Health Education | Optional (encouraged) | Regular推送 of rehabilitation education and health knowledge content on lifestyle adjustments after lumbar disc herniation surgery; usage logged | Number of educational materials opened per patient over 3-month follow-up period (mean ± SD) |
| Doctor-Patient Interaction | Optional | Patients can communicate with designated doctors daily via text or voice messages | Number of messages exchanged per week (mean ± SD) |
| Sharing Area | Optional | Patients can share experiences based on their disease and cultural backgrounds | Qualitative assessment only; not quantified for this analysis |
| Daily Check-in | Mandatory | Patients upload progress reports (text and optionally video) on their exercise execution; check-in data enables nurses to adjust exercise prescriptions and send remote reminders if exercises are missed | Daily check-in completion rate (same as above); system automatically triggers nurse follow-up call if check-in rate <70% for two consecutive weeks |
| Smart Questionnaire | Mandatory (at scheduled time points) | Facilitates data collection through automated patient surveys administered at discharge, 1 month, and 3 months post-discharge | Completion rate at scheduled time points (reported as percentage in Section 3.1) |
| One-click Help | Mandatory (always available) | Provides 24/7 access to on-duty doctors for immediate assistance and guidance for patients experiencing sudden pain or muscle weakness | Usage frequency (number of activations) reported descriptively |

**Note:** Attribute definitions: "Mandatory" indicates the module is automatically activated for all patients in the intervention group and is required for protocol adherence; "Optional" indicates the module is available for patient-initiated use, and non-use does not constitute protocol deviation (usage is logged and reported descriptively). Quantitative adherence metrics reported in this table correspond to the data presented in Section 3.8 (Patient engagement with the digital intervention). AI monitoring system validation performance (sensitivity, specificity, accuracy, and kappa) is reported separately in **Table S2**.

**Table S2** Patient engagement metrics for the digital intervention over 3‑month follow‑up

| **Module** | **Attribute** | **Adherence metric** | **Value (mean ± SD or n/N, %)** | **Interpretation** |
| --- | --- | --- | --- | --- |
| Daily Reminders | Mandatory | Daily check-in completion rate | 84.6% ± 11.2% | High adherence; 42/50 (84%) patients achieved ≥80% check-in rate |
| Functional Exercise | Mandatory | AI monitoring usage frequency | 16.3 ± 6.1 sessions/patient | 85.2% of exercise sessions were AI‑monitored |
| Functional Exercise | Mandatory | Exercise video viewing count | 22.4 ± 8.7 plays/patient | Patients viewed videos on 89.6% of exercise days |
| Smart Questionnaire | Mandatory | Scheduled completion rate | 100% at 1 month; 98% at 3 months | Near‑perfect data capture for outcome assessments |
| One‑click Help | Mandatory | Activation frequency | 9 activations by 6 patients (12.0%) | All resolved within 24h; no emergent referrals |
| Health Education | Optional | Materials accessed | 12.5 ± 5.3 materials/patient | 84.0% accessed ≥1 material/week in month 1; 56.0% in month 3 |
| Doctor‑Patient Interaction | Optional | Messages exchanged/week | 2.1 ± 1.4 messages/week | Wide variability (range 0.3–6.8); indicates personalized use |
| Sharing Area | Optional | Posts generated | 67 total posts by 23 patients (46.0%) | Moderate engagement; primarily peer encouragement content |

**Table S3** AI movement monitoring system validation performance stratified by exercise type

| **Exercise type** | **N (videos)** | **Sensitivity (%, 95% CI)** | **Specificity (%, 95% CI)** | **Accuracy (%, 95% CI)** | **PPV (%, 95% CI)** | **NPV (%, 95% CI)** |
| --- | --- | --- | --- | --- | --- | --- |
| Five-point support | 100 | 87.2 (82.0–91.5) | 90.5 (86.0–94.2) | 89.6 (85.8–93.0) | 88.3 (83.6–92.4) | 89.5 (85.0–93.4) |
| Three-point support | 100 | 84.8 (79.2–89.6) | 88.9 (84.0–93.0) | 87.2 (82.6–91.2) | 86.5 (81.2–91.0) | 87.3 (82.5–91.6) |
| Plank | 100 | 90.4 (85.5–94.4) | 91.8 (87.4–95.4) | 91.2 (87.5–94.5) | 90.7 (86.0–94.6) | 91.6 (87.2–95.2) |
| Supine unilateral knee flexion | 100 | 84.0 (78.0–89.2) | 87.6 (82.4–92.0) | 86.0 (81.0–90.4) | 85.1 (79.6–89.8) | 86.5 (81.2–91.0) |
| Supine bilateral knee-to-chest | 100 | 83.6 (77.6–88.8) | 88.2 (83.0–92.6) | 86.4 (81.4–91.0) | 85.3 (79.8–90.0) | 86.7 (81.6–91.4) |
| Standing lunge | 100 | 80.8 (74.4–86.4) | 85.6 (80.0–90.4) | 83.4 (78.0–88.4) | 82.0 (75.8–87.6) | 84.5 (78.8–89.6) |
| Overall | 500 | 85.6 (82.1–88.7) | 89.2 (86.0–92.1) | 87.3 (84.9–89.5) | 86.3 (82.8–89.4) | 88.6 (85.3–91.5) |

**Note:** PPV, positive predictive value; NPV, negative predictive value; CI, confidence interval. Physician consensus reference standard (≥2 of 3 rehabilitation physicians, each with ≥5 years of experience in spinal rehabilitation) was used as the comparator. The validation dataset comprised 500 videos (100 per exercise type) collected from 20 healthy volunteers who were not involved in the training phase. Physicians were blinded to the algorithm's classifications. The algorithm was configured to detect form errors based on predefined joint angle criteria (e.g., hip angle deviation >15 from neutral for plank; knee extension <160 for standing lunge).
